# Tuberculosis detection from raw sputum samples using Au-electroplated screen-printed electrodes as E-DNA sensor

**DOI:** 10.3389/fchem.2022.1046930

**Published:** 2022-11-21

**Authors:** M. N. Sharif, S. Taufiq, M. Sohail, S. R. Abbas

**Affiliations:** ^1^ Biosensors and Therapeutics Lab, School of Interdisciplinary Engineering and Sciences (SINES), Islamabad, Pakistan; ^2^ Department of Industrial Biotechnology, Atta Ur Rahman School of Applied Biosciences (ASAB), Islamabad, Pakistan; ^3^ Department of Chemistry, School of Natural Sciences (SNS), National University of Sciences and Technology (NUST), Islamabad, Pakistan

**Keywords:** IS-6110, mtb detection, tuberculosis, screen printed electrode (SPE), electrochemical biosensing

## Abstract

Tuberculosis (TB) remains a leading cause of death globally, especially in underdeveloped nations. The main impediment to TB eradication is a lack of efficient diagnostic tools for disease diagnosis. In this work, label free and ultrasensitive electrochemical DNA biosensor for detecting *Mycobacterium tuberculosis* has been developed based on the electrodeposition of gold nanoparticles on the surface of carbon screen-printed carbon electrode (Zensors) for signal amplification. Particularly, screen-printed electrodes were modified by electrochemical deposition of Au to enhance the conductivity and facilitate the immobilization of ssDNA probes *via* Au-S bonds. The electrochemically modified SPEs were characterized using Scanning electron microscopy/Energy Dispersive X-Ray Analysis (SEM/EDX) and X-Ray Diffraction (XRD). Cyclic voltammetry (CV) and differential pulse voltammetry (DPV) techniques were used to investigate the DNA hybridization between single-stranded (ssDNA) probe and target DNA (tDNA). Under the ideal conditions, DPV exhibited a correlation coefficient R2 = 0.97, when analyzed with different tDNA concentrations. The proposed DNA biosensor exhibits a good detection range from 2 to 10 nm with a low detection limit of 1.91 nm, as well as high selectivity that, under ideal conditions, distinguishes non-complementary DNA from perfectly matched tDNA. By eliminating the need for DNA purification, this work paves the path for creating disposable biosensors capable of detecting DNA from raw sputum samples.

## Graphical Abstract

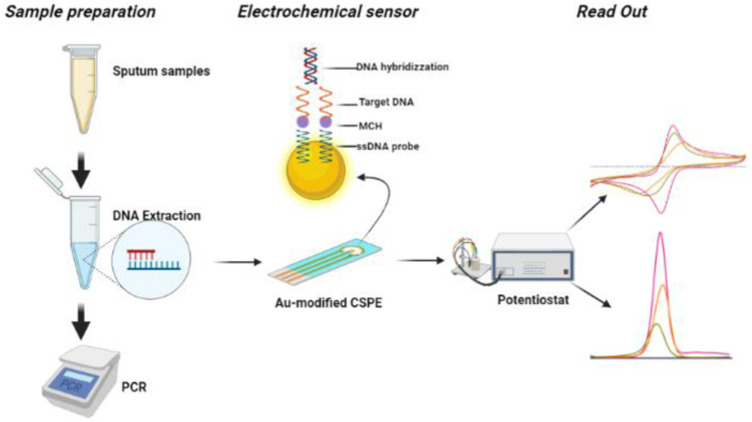

## Introduction

Tuberculosis (TB) is a life-threatening bacterial infection caused by *Mycobacterium tuberculosis*, which spreads through the air when a person with tuberculosis coughs or sneezes. According to World Health Organization (WHO) estimates, there are approximately 10 million new cases per year, of which 1.5 million succumb to death, and it is one of the top ten global causes of mortality (WHO Global tuberculosis report 2021) (Sypabekova et al., 2019). This pathogen is an obligate aerobic bacterium that causes a bacterial proliferative phase, followed by an immune reaction against the bacteria that pushes TB into a latent phase without symptoms. Latent and early-stage TB patients are challenging to diagnose and represent a reservoir of the disease in the community (Getahun et al., 2015). Early diagnosis of active infection is essential to treat tuberculosis and limit its spread effectively. Chest X-ray, sputum smear, culture, immunology, molecular biology, and other procedures are currently utilized for the clinical detection of TB (N’guessan et al., 2016; Chen et al., 2018). The procedures of chest X-ray and sputum smear cannot be used for the early detection of patients as this method requires four to 6 weeks to yield results (Achkar and Prados-Rosales, 2018). When cartridge-based molecular assay Xpert MTB/RIF was introduced in 2010, a paradigm shift occurred in TB diagnostics due to its enhanced sensitivity for smear-negative disease. However, same-day reporting of test findings is sometimes not possible because of delays in sample transport to reference labs (Kwak et al., 2013). Therefore, accurate point-of-care testing for the detection of active disease remains a top priority.

**TABLE:** 1 Comparison of the present work and other reported electrochemical sensing technique for the detection of *M. tuberculosis*.

Electrode	Probe	Limit of detection (LOD)	References
ITO	DNA	0.10 × 10^−15^M	Mogha *et al.* (2018)
GCE	DNA	5.45 × 10^−13^M	Liu *et al.* (2014)
SPE	DNA	8.95 × 10^−13^M	Mat Zaid *et al.* (2017)
SPE	DNA	7.85 × 10^−7^M	Mohamad *et al.* (2017)
SPCE	DNA	170 × 10^–12^ M	Torres-Chavolla and Alocilja, (2011)
Au-SPE	DNA	1.9 nm	This Work

Electrochemical detection methods have garnered significant scientific interest due to their portability, low cost, rapid response, noninvasive nature, high selectivity and sensitivity, excellent reproducibility and stability, and ease of use (Ashraf et al., 2021; Tran et al., 2021). An electrochemical biosensor can quantify target DNA concentration by detecting changes in electrochemical signal induced by the hybridization reaction between the capture probe and target DNA (tDNA) on the electrode’s surface. When compared to other traditional approaches, the electrochemical DNA biosensor has higher sensitivity and portability (Ma et al., 2022). In recent years, different nanomaterials have been used in sensing technology to increase the sensitivity of biosensors. Nanomaterials are best known for their large effective surface area, which enables the immobilization of a more significant number of bioreceptors on the surface of the working electrode (Holzinger et al., 2014). Because of their unique properties, including a large specific surface area, high conductivity, and biocompatibility, gold nanoparticles (AuNPs) are widely used as immobilizing substrates for biomolecules in the fabrication of DNA biosensors (Pilehvar et al., 2017; Duan et al., 2018).

Among the various types of electrodes used as transducers in electrochemical biosensors, screen-printed electrodes (SPE) have attracted great interest since they permit sensitive and selective analysis at a low cost, which enables their use as disposable devices, and are suitable for the analysis of low sample volumes (Yamanaka et al., 2016). Furthermore, SPEs have the benefit of being easily mass-produced, with no prior tedious preparation stages or necessity for highly experienced individuals (Li et al., 2017). Screen-printing technology also has other interesting advantages, such as electrodes with different spacings that can be used for miniaturized devices and the ability to use small sample volumes (Yáñez-Sedeño et al., 2018). Screen-printed electrodes can be used in conjunction with basic, portable electrochemical equipment, and the samples can be analyzed using different electroanalytical methods such as Cyclic voltammetry, Differential Pulse Voltammetry, and EIS (Del Torno-De Román et al., 2016; Sgobbi et al., 2016).

In this study, a DNA-based electrochemical sensor platform for the early detection of tuberculosis from purified DNA samples and sputum was developed using commercially available screen-printed electrodes from Zensors. These SPEs were modified with gold using an electrodeposition technique. A DNA probe has been used to detect the insertion element IS6110, a mobile genetic element utilized to detect tuberculosis. IS6110 is a 1354-bp repeated insertion sequence found in 1–20 copies per cell. Due to its high amount of replication throughout the genome, it is regarded as one of the gold-standard biomarkers for MTB detection (Chu et al., 2019; Eloi et al., 2020). Differential pulse voltammetry (DPV) and Cyclic voltammetry were employed to evaluate the performance of the sensor platform.

## Experimental section

### Reagents and materials

Carbon screen-printed electrodes (CSPEs) were purchased from Zensors. All solutions were prepared in deionized (DI) water. Potassium Ferricyanide K_3_Fe(CN)_6_, Potassium Chloride (KCl), 6-Mercapto-1-Hexanol (MCH), HAuCl_4_, Dithiotrietol (DTT), Triethylamine (TEA) were purchased from Sigma–Aldrich and used as received. The SH modified probe used in the study was purchased from Penicon.

### DNA probe reduction

Thiol-modified oligos were given in a disulfide-protected form to minimize their oxidation potential. Dithiothreitol (DTT) was used to activate the thiol bond in accordance with a Sigma Aldrich-recommended technique for disulfide bond reduction (-SH). To activate the thiol bonds, up to 1 mg of oligo was reconstituted in 200 µl of 2% TEA (triethylamine) and 50 mm DTT and incubated for 10–15 min at room temperature. Afterward, DTT was eliminated by acetone precipitation. After this, a 5:1 acetone-oligo solution (2% LiClO4 w/w in acetone) was prepared and cooled at −20°C for 15 min. The prepared solution was centrifuged for 10–15 min at 13,000 rpm. The supernatant was collected, and the sample was vacuum-dried to eliminate any remaining acetone. To remove LiClO4 and other salts, the sample was washed with 2–3 ml n-butanol, centrifuged, then washed again to remove the butanol supernatant. For future usage, the pellet was resuspended in TE buffer and kept at 20°C.

### Clinical samples preparation

The expectorated sputum samples is the most commonly used diagnostic specimen for tuberculosis diagnosis. Sputum samples were collected from TB patients at the NIH’s National Reference Lab for TB (NRL). Sputum was collected in properly capped, wide-mouthed 50 ml clear containers. The N-acetyl-l-Cysteine (NALC)-NaOH method was used to disinfect the sample. NALC serves as a mucolytic agent, releasing acid-fast bacilli (AFB) from sputum, while NaOH acts as a decontaminant, destroying the microorganisms.

### Genomic DNA extraction

DNA was extracted from raw sputum samples using ready-to-use extraction kits. Sputum bacterial sediment was resuspended in 90 µL of PBS solution and vortexed. 10 µL of a 25 mg/ml lysozyme solution was added to the suspended solution. The solution was then maintained at 37°C for 10 min until it turned transparent. Then, 10 µL of proteinase K solution was added, and the mixture was maintained at 56°C for at least 30 min. The sample was then placed into the extraction column, followed by adding 100 µL of DNA binding buffer. For the binding of gDNA to the column matrix, the tube was screw-capped and centrifuged at 1,000 × g for 5 min. After adding 300 µL of wash buffer to the spin column, it was centrifuged at 1000 × g for 1 min. After adding 300 µL of wash buffer to the spin column, it was centrifuged at 1000 × g for 1 min, followed by adding 100 µL elution buffer.

The DNA isolated from eight sputum samples was then amplified using a Bio-RAD T100 thermal cycler. Forward (5′-AGA​AGG​CGT​ACT​CGA​CCT​GA-3′) and reverse (5′-GAT​CGT​CTC​CGG​CTA​GTG​CAT-3′) primers specific for the amplification of IS6110 region of MTB were used.

### SPE surface modification and characterization

Carbon screen-printed electrodes procured from Zensors were electroplated with gold to improve the conductivity and the immobilization of the ssDNA probe on the surface of the working electrode. The gold nanoparticles were electrodeposited onto the surface of the working electrode by cycling the potential from −1.4V to 0 V at 50 mV s^−1^ (6cycles) using cyclic voltammetry. The carbon working electrode was modified in 6 mm HAuCl4 containing 0.1 M KCL solution. In the next step, modified SPEs were incubated with 4 µL of ssDNA probe for 1 h, followed by incubation with 1 mm MCH for another 45 min. MCH was used to block the non-specific binding at the electrode surface and change DNA orientation. This assembly of the ssDNA probe and MCH on a gold surface significantly increased DNA hybridization. After this, Au-SPE/ssDNA/MCH functionalized electrode was incubated with target DNA for 1 h to allow the hybridization of DNA, and changes in the electrochemical signal were detected using CV and DPV. The morphological properties of screen-printed electrodes were analyzed and validated using Scanning electron microscopy (SEM) (Tescan Vega 3). Scanning was carried out at a voltage of 20 kV, with a maximum magnification of ×65,000 and a working distance of 5.07 mm. Moreover, it was characterized through X-ray diffraction (XRD) to determine the crystallographic structure of electrodeposited gold. XRD of Au-modified SPE was performed using the STOE Powder Diffractometer θ-θ (STOE Inc. Germany).

### Electrochemical detection of *Mycobacterium tuberculosis*

**FIGURE 1:**
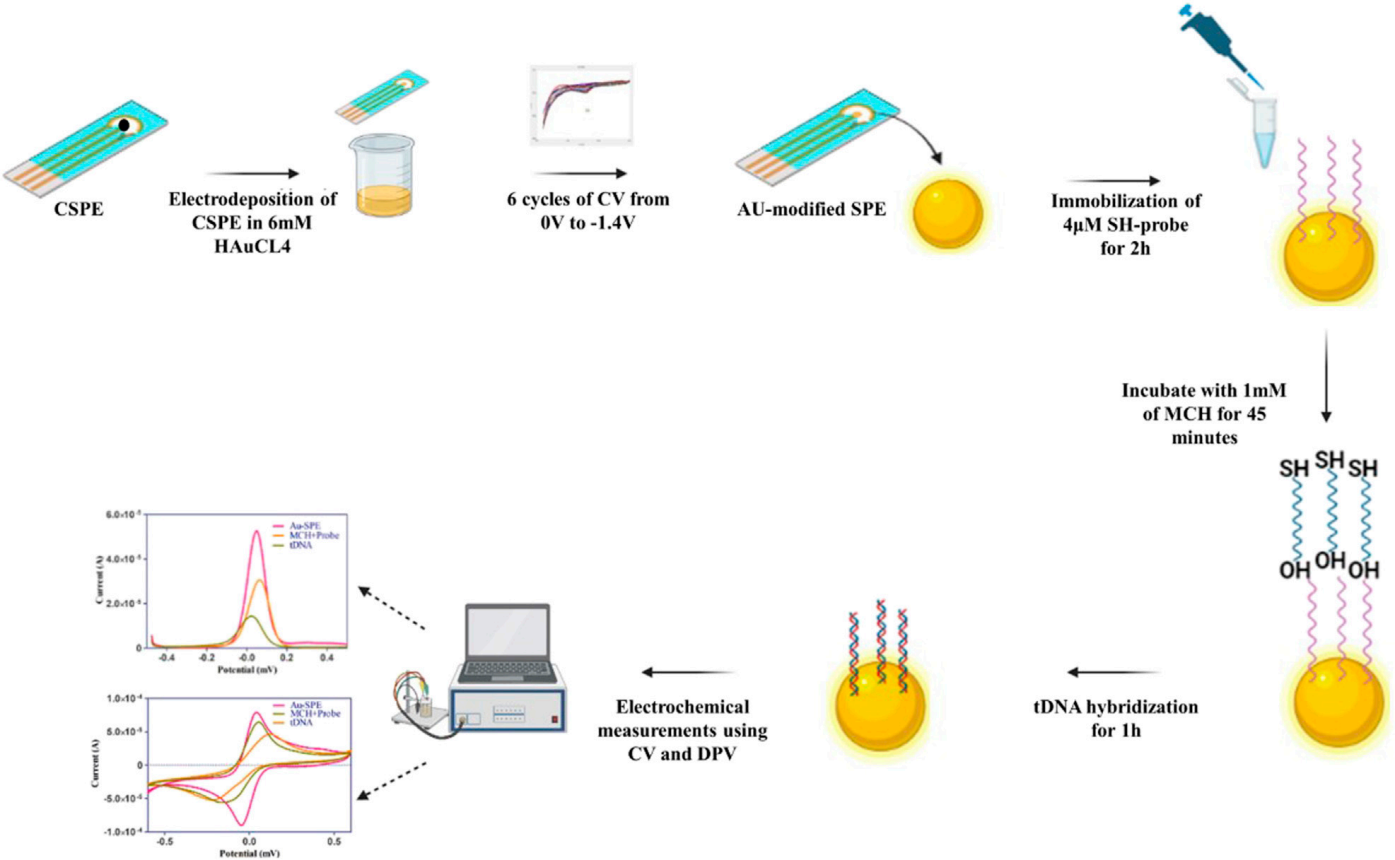
Schematic illustration of stepwise modification of screen-printed electrode for the detection *Mtb* DNA.

The electrochemical analysis was performed on Gamry Interface 1010 B. The change in the electrochemical signals was studied using Cyclic Voltammetry (CV) and Differential Pulse Voltammetry (DPV) techniques. The stability of the electrodeposited gold SPEs were investigated by increasing the scan rates (20 mV/s, 40 mV/s, 60 mV/s, 80 mV/s, and 100 mV/s). All electrochemical measurements were performed at room temperature. CV with the potential range of −0.8 V–0.8 V at a scan rate of 100 mV s^−1^ and DPV with the potential range of −0.5 V–0.5 V at a scan rate of 25 mV s^−1^ in 5 mm K^3^ [Fe(CN)]^6^ containing 0.1 M KCl as supporting electrolyte was performed. The stepwise modification of Screen-printed electrodes with Au for the detection of Mtb is depited in Figure 1.

## Results and discussion

### Characterization of carbon and Au-modified SPEs

**FIGURE 2:**
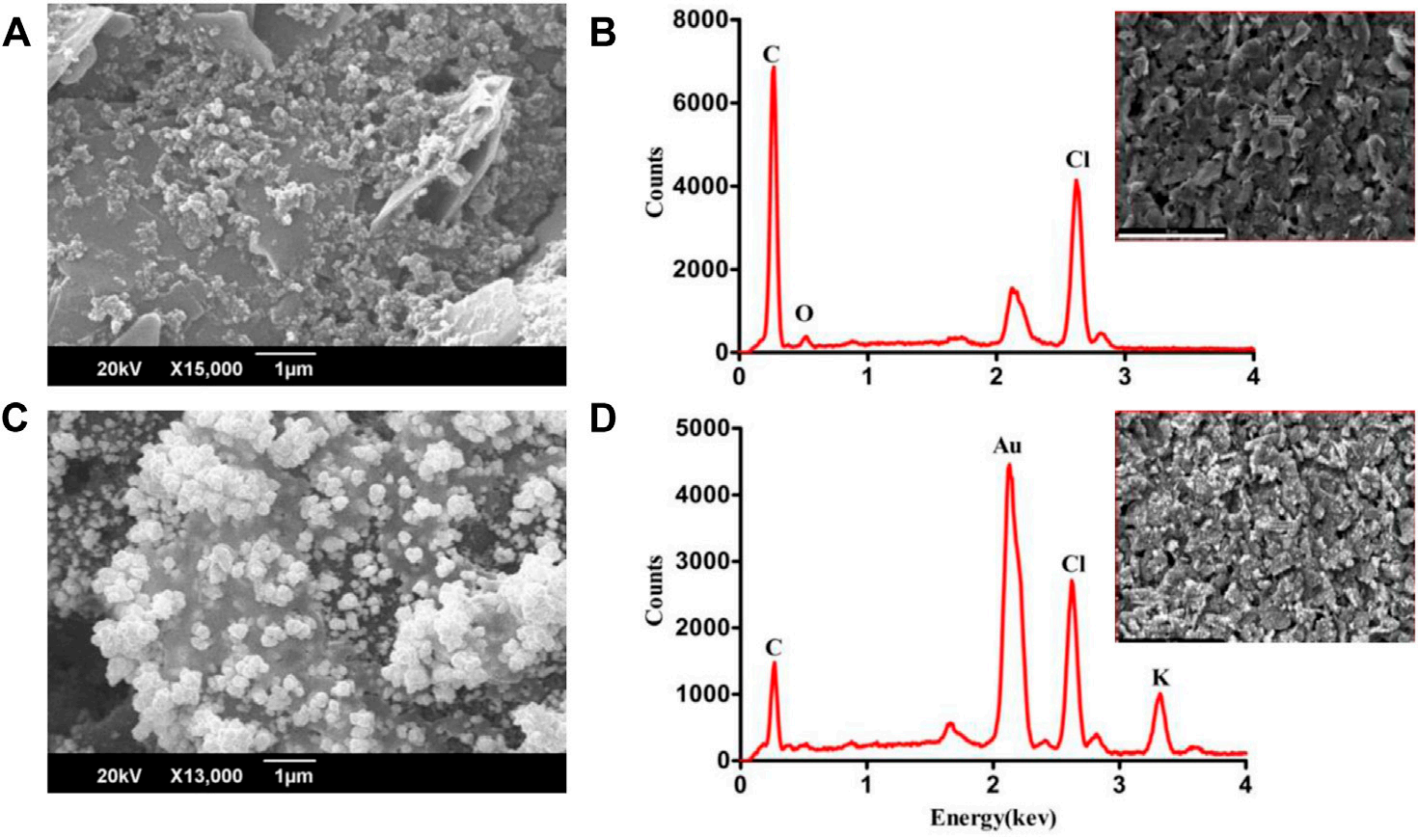
SEM and EDX analysis of carbon SPE **(A,B)** and electrodeposited gold SPE **(C,D)**.

The surface morphology of bare and electrodeposited-Au screen-printed electrodes was analyzed using the scanning electron microscope. The SEM investigation of bare carbon SPE revealed irregularly shaped graphite flakes (Figure 2A), randomly oriented inside the original ink. The SEM images of Au-modified SPEs in Figure 2B reveal successful deposition of Au, while the electrodeposition was performed by applying a voltage ranging from 0 V to −1.4 V. Figures 2B,D depict the EDX spectra of carbon SPE and Au-modified SPE, respectively. The presence of C in the EDX spectrum indicates the utilization of graphite flakes and carbon ink for the deposition of bare SPE. The existence of gold peaks in the EDX spectrum indicates that Au was successfully electrodeposited on CSPE. The electrochemical characteristics of modified screen-printed electrodes are principally influenced by the morphology and shape of deposited nanoparticles due to the electroactive regions (Carbone et al., 2017). The observed atomic percentage C and Au in EDX analysis of bare carbon SPE and electrodeposited Au was 83% and 55%, respectively.

**FIGURE 3:**
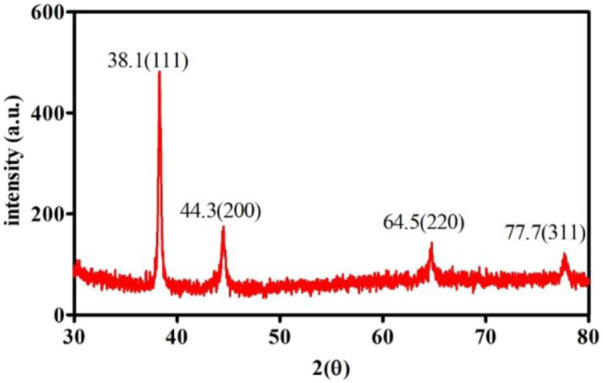
XRD analysis of Au-modified SPE.

Furthermore, the crystallinity of synthesized AuNPs was confirmed *via* the X-ray diffraction (XRD) technique, and Figure represents XRD patterns of the synthesized gold nanoparticles. Four distinct peaks can be observed in Figure 3 at 2θ = 38.1.44.3, 64.5, and 77.7. All four peaks were consistent with standard Bragg reflections (111), (200), (220), and 311) of face center cubic (fcc) lattice. The 38.1 peak (intense diffraction) indicates that the preferred growth orientation of zero-valent gold was fixed in the 111) direction. This XRD pattern indicates that the sample is pure Au nanocrystals. These peaks match with Joint Powder Diffraction Standards (JCPDS) 00-004-0784. The results show that these are molecular-sized solids formed with a repeating 3D pattern of atoms or molecules with an equal distance between each part (Khalil et al., 2012; Krishnamurthy et al., 2014).

### Electrochemical characterizations

**FIGURE 4:**
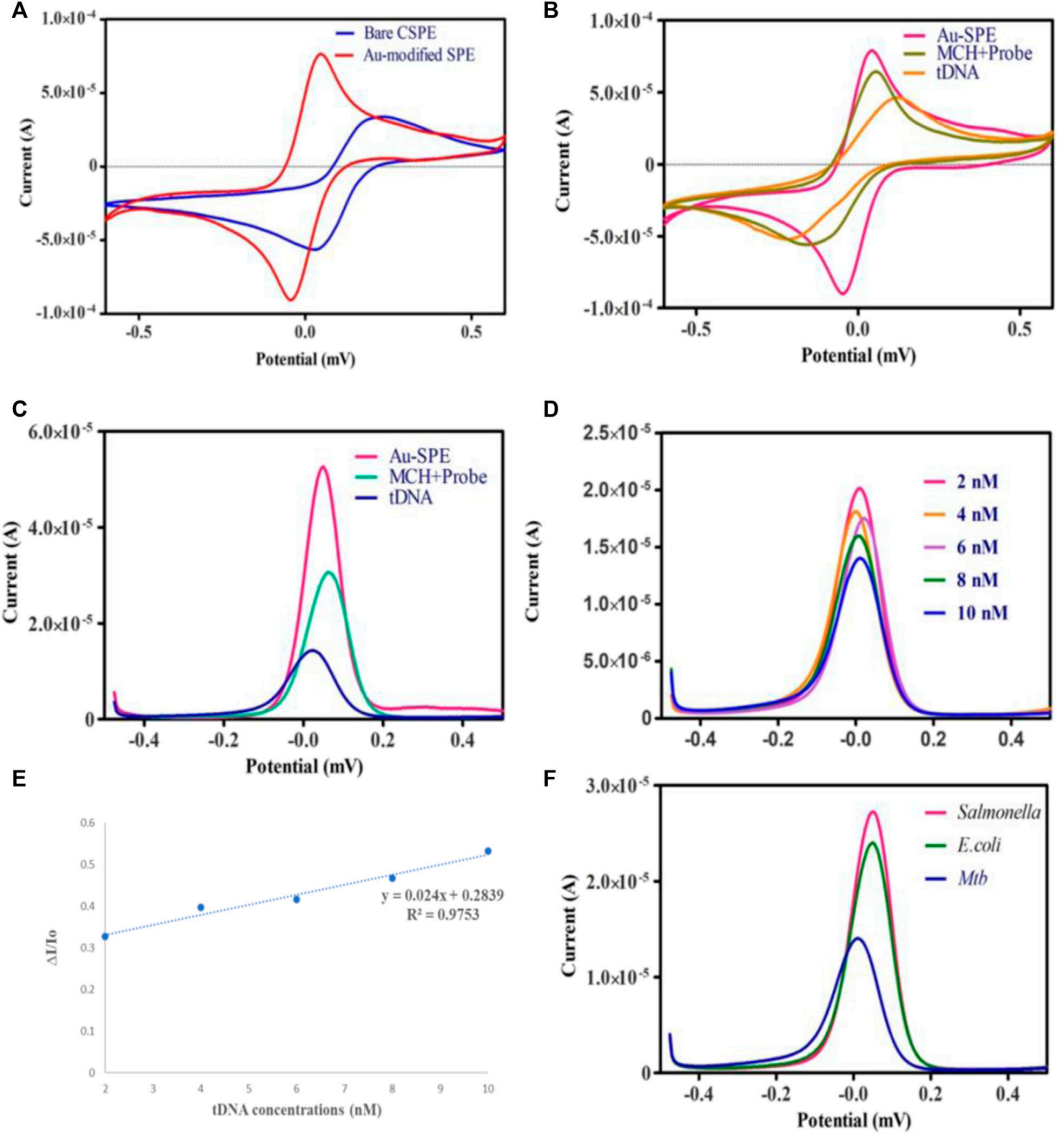
**(A)** Cyclic voltammetry of bare Carbon SPE and electrodeposited Au-SPE. **(B,C)** CV and DPV analysis of Au-modified SPE functionalized with ssDNA probe/MCH and tDNA in 5 mm K_3_ [Fe (CN)_6_] and 0.1 M KCl. **(D)** DPV analysis of different *Mtb*-tDNA concentrations ranging (2nm–10 nm) on modified SPE to check the analytical performance of biosensor. **(E)** Linear correlation of current response against the different concentration of target DNA. **(F)** Specificity of biosensor against *Salmonella*, *E. coli* and *Mtb*.

Cyclic voltammetry (CV) and differential pulse voltammetry (DPV) were employed to assess the feasibility of the developed electrochemical biosensor in 0.1 M KCl containing 5 mm [Fe(CN)^6^]. The electrochemical active surface area of carbon screen printed electrode was 0.035 cm^2^. But after the SPE surface modification with Au the electrochemically active surface area of Au-SPE was found to be 0.14 cm^2^ by using ECSA = Cdl ^(double layer capacitance)^/Cs ^(specific capacitance)^ formula. When carbon SPEs were functionalized with Au, they exhibited increased electrode conductivity compared to bare SPEs, which can be observed in Figure 4A, allowing for effective electron transport on the surface of the electrode (Shi et al., 2018). The electrochemical signal was decreased (Figures 4B,C) when ssDNA probe was immobilized on the surface of Au-modified SPE due to the electrostatic repulsion between negatively charged electrolyte solution and ssDNA probe (Niu et al., 2017). After treating the surface of Au-SPE/ssDNA with 6-mercapto-1-hexanol (MCH) to block the non-specific binding sites, the current is lowered further. This happened on the surface of the electrode due to the formation of an insulating MCH layer which hinders electron transfer (Wang et al., 2021). The electrochemical event implies hybridization between two oligonucleotide strands, which results in increased electrostatic repulsion, thus decreasing the height of the cathodic and anodic peaks (Figures 4B,C). Due to the creation of an electron transport barrier, the current values decrease as the number of cDNA conjugates at the transducer interface increases. Higher concentrations of cDNA conjugates at the transducer interface result in lower current values due to the creation of a barrier to the electron transport (Shamsipur et al., 2020).

Under optimum conditions, the electrochemical performance of the biosensor was evaluated at several different tDNA concentrations to check the sensitivity of the biosensor. Figure 4D demonstrates that the signal response is highly dependent on the concentration of target DNA. Increasing the target DNA concentration from 2 nm to 10 nm decreases the intensity of the current. In this study, the ssDNA/MCH/Au-SPE was incubated with various concentrations of the target DNA. The DNA biosensors rely on the interactions of free guanine of probe ssDNA with their complementary bases of tDNA during the DNA hybridization, as a result of which less free guanine is oxidized (Rashid and Yusof, 2017). Therefore, the hybridization process results in a lower oxidation signal of guanine. In addition, a significant linear relationship (R2 = 0.9753) exists between the tDNA concentration and peak current for modified SPEs (Figure 4E). Moreover, the Limit of Detection (LOD) for the proposed biosensor is 1.9 nm, estimated by the 3σ rule. The obtained LOD was compared to the different electrochemical techniques used for the detection of tuberculosis (Table 1).

**FIGURE 5:**
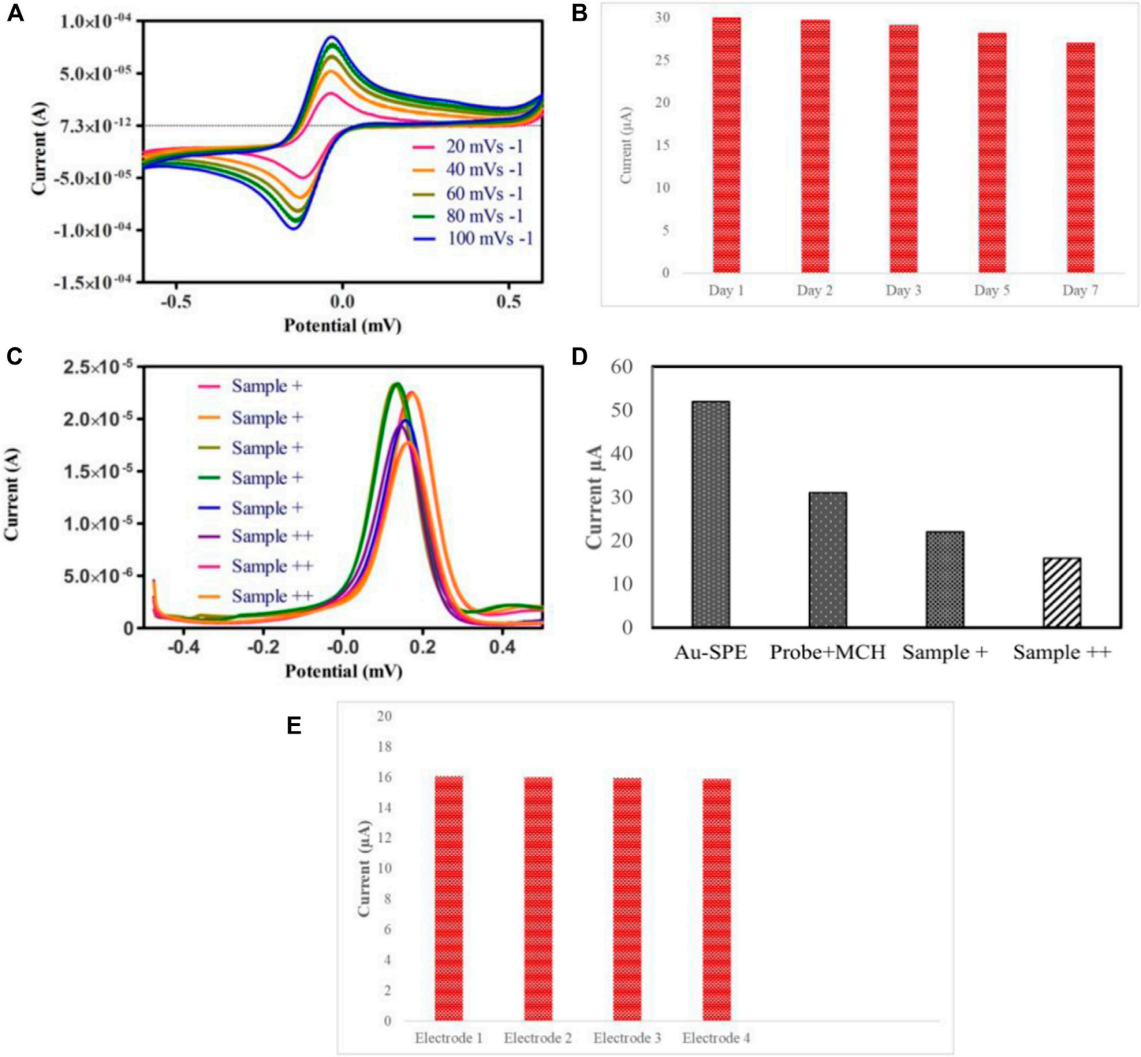
**(A)** CV voltammogram of Au-modified SPE in the 5 mm K_3_ [Fe (CN)_6_] and 0.1 KCl solution at various scan rates 20, 40, 60, 80, 100 mVs^−1^. **(B)** Stability investigation of the fabricated biosensor modified with ssDNA probe and MCH. **(C)** DPV analysis of raw sputum samples divided into sample+ and sample++ based on bacillary loads **(D)** Histogram of electrochemical response of the clinical sputum samples grouped according to the bacillary load. **(E)** Reproducibility of biosensor.

Furthermore, specificity analysis was done by testing the biosensor against the DNA of different bacterial species, such as E. *coli* and *Salmonella typhimurium*. All the bacterial DNA sequences were prepared at a concentration of 4 nm, and the process was carried out under ideal conditions. It can be observed in Figure 4F that the decrease in the peak current in the case of E. *coli* and *Salmonella typhimurium* was minimal compared to Mtb DNA. The binding of probe DNA’s guanine bases to their complementary bases lowered the number of redox-active guanine groups available for oxidation, hence decreasing the peak current, which occurred in the case of Mtb DNA. As depicted in Figure 4F, the DNA biosensor could distinguish between complementary and non-complementary DNA sequences based on the response of the DPV curves. These findings verified the excellent specificity of the fabricated biosensor. A series of CV analyses of SPEs functionalized with Au at different scan rates of 20, 40, 60, 80, and 100 mV/s in the potential range of −0.6V to 0.6 V revealed the effect of scan rate on the current response of the modified electrodes (Figure 5A).

A linear relationship between the scan rate and the oxidation and reduction peak currents indicates that adsorption is controlled on the surface of the modified electrode. A linear relation between scan rate and reduction and oxidation peak currents suggests that the adsorption is controlled on the surface of modified SPEs due to the mass transfer phenomenon at the solution-electrode interface (Khan et al., 2018).

### Stability and reroducibility of biosensor

The most critical parameter for validating the practical application in real-time for bacterial detection is the stability and reproducibility of the DNA biosensor. To test the biosensor’s stability, a set of Au-modified SPEs functionalized with ssDNA probe/MCH were stored at 4°C for 7 days, and DPV measurements were taken. The current signal of the biosensor after 7 days of storage at 4°C in the refrigerator is 90% of the original signal, suggesting that the sensor has good stability, as depicted in Figure 5B. Subsequently, we further studied the reproducibility of the biosensor by hybridizing same concentration of target DNA on different electrodes. The reproducibility of the biosensor was explored by measuring the change in peak currents, as depicted in Figure 5E. The results suggest that the Au-modified electrode exhibited good stability for detecting target DNA.

### Clinical samples

Eight clinical sputum samples were used to determine the practicality of the fabricated biosensor, which were first confirmed by smear microscopy and Gene Xpert. All the sputum samples were grouped according to their bacillary load as + and ++ (higher bacillary load). It can be observed in Figures 5C,D that samples containing higher bacillary load hindered the electron transfer, thus resulting in a significant decrease in the current as compared to + samples.

## Conclusion

A highly sensitive and selective electrochemical DNA biosensor was fabricated to detect IS6110 of *Mycobacterium tuberculosis*. The carbon SPEs were modified with Au using the electro-deposition technique to improve the conductivity of the working electrode. SEM/EDX and XRD confirmed the SPEs surface functionalization. CV and DPV techniques were used to investigate the feasibility of the biosensor. To check the sensitivity, different concentrations of target DNA were tested, ranging from 2 nm to 10 nm with a detection limit of 1.9 nm. The proposed biosensor was simple to design and showed high sensitivity and selectivity for detecting purified DNA and clinical tuberculosis samples. Considering these benefits, the proposed biosensor can be a promising tool for rapidly detecting tuberculosis and controlling TB prevalence.

## Data Availability

The original contributions presented in the study are included in the article/Supplementary Material, further inquiries can be directed to the corresponding author.
